# Examining the Impact of the National Institutes of Health Public Access Policy on the Citation Rates of Journal Articles

**DOI:** 10.1371/journal.pone.0139951

**Published:** 2015-10-08

**Authors:** Sandra L. De Groote, Mary Shultz, Neil R. Smalheiser

**Affiliations:** 1 University Library, University of Illinois at Chicago, Chicago, IL, United States of America; 2 Savitt Medical Library, University of Nevada School of Medicine, Reno, NV, United States of America; 3 Department of Psychiatry, College of Medicine, University of Illinois at Chicago, Chicago, IL, United States of America; Max Planck Society, GERMANY

## Abstract

**Purpose:**

To examine whether National Institutes of Health (NIH) funded articles that were archived in PubMed Central (PMC) after the release of the 2008 NIH Public Access Policy show greater scholarly impact than comparable articles not archived in PMC.

**Methods:**

A list of journals across several subject areas was developed from which to collect article citation data. Citation information and cited reference counts of the articles published in 2006 and 2009 from 122 journals were obtained from the Scopus database. The articles were separated into categories of NIH funded, non-NIH funded and whether they were deposited in PubMed Central. An analysis of citation data across a five-year timespan was performed on this set of articles.

**Results:**

A total of 45,716 articles were examined, including 7,960 with NIH-funding. An analysis of the number of times these articles were cited found that NIH-funded 2006 articles in PMC were not cited significantly more than NIH-funded non-PMC articles. However, 2009 NIH funded articles in PMC were cited 26% more than 2009 NIH funded articles not in PMC, 5 years after publication. This result is highly significant even after controlling for journal (as a proxy of article quality and topic).

**Conclusion:**

Our analysis suggests that factors occurring between 2006 and 2009 produced a subsequent boost in scholarly impact of PubMed Central. The 2008 Public Access Policy is likely to be one such factor, but others may have contributed as well (e.g., growing size and visibility of PMC, increasing availability of full-text linkouts from PubMed, and indexing of PMC articles by Google Scholar).

## Introduction

In 2004, the National Institutes of Health (NIH) requested that funded investigators voluntarily deposit an electronic version of their final manuscript in PubMed Central (PMC), upon acceptance for publication [1]. On April 7, 2008, the NIH announced a Public Access Policy, to "ensure that the public has access to the published results of NIH funded research" [2] and to "help advance science and improve human health", which required that all peer-reviewed articles resulting from research funded by the NIH are deposited in and publicly accessible from PMC within 12 months of publication [3,4]. With the 2008 policy, PMC submission is a requirement of funding. The present study seeks to determine if free availability through PMC increases the scholarly impact of research. Specifically, we examine whether articles published after the release of the 2008 NIH Public Access Policy (PAP) and archived in PMC showed a boost in scholarly impact relative to articles that were not archived in PMC. Citation rates, both yearly and cumulative over five years, were analyzed as a measure of scholarly impact.

### Background

PMC, launched in 2000, is a "free archive of biomedical and life sciences journal literature" managed by the U.S. NIH National Library of Medicine as part of its "legislative mandate to collect and preserve the biomedical literature" [5]. PMC is related to the larger PubMed database, but is, in fact, a separate system. PubMed includes citations to journal articles from the MEDLINE database and other life sciences journals. It currently has over 24 million citations to journal articles. PubMed is not a full-text database although it offers link-outs from citations to full-text provided through publishers (often accessible through institutional subscriptions), as well as freely available full-text articles, including those on its sister site, PMC. PMC is a repository of freely available full-text journal articles and currently contains over 3.3 million articles [6].

There are two main ways in which an article can be archived in PMC: 1) participating publishers may archive their entire journal(s) in PMC and thus all the articles published in those journals would appear in PMC; 2) individual articles not in such journals, yet falling within the NIH mandate, should be deposited in PMC by their author or by the journal [7]. An examination of the NIH Manuscript Submission (NIHMS) System Statistics illustrates that the number of manuscripts submitted to the NIHMS system dramatically increased with the implementation of the NIH Public Access Policy. Currently, the number of manuscripts submitted to the system averages over 5,500 per month [8]. As of January 2013, the NIH estimated that the 2008 Public Access Policy’s compliance rate was approximately 75% [9]. In addition to the above methods for depositing content in PMC, there are collaborative efforts on an international level to have organizations in other countries use PMC as an archive [10]. This is referred to as PMC International (PMCI) which currently has two centers, Europe PMC and PMC Canada. Both deposit articles into PMC which have funding from sources equivalent to the NIH in the U.S.

The likely ways that users are currently accessing PMC articles online include: a) searching PMC directly; b) searching PubMed and linking from the PubMed citation to the PMC full-text article; or c) by searching Google Scholar and linking from the Google Scholar citation to the PMC full-text article. Both Google Scholar and PubMed typically include links to the publisher version of the article on the publisher website as well as a link to the PMC full-text version. Full-text access to the publisher version of the article is usually dependent on fee-based subscriptions, either institutional or individual, unless the journal is open access or the publisher has made journal content freely available after an embargo period. Approximately 14% of PubMed citations link to a PMC article. In 2013, approximately 3 million PubMed searches were performed on a daily basis through the PubMed Web site and an additional 3 million with scripts (application programming interfaces) [11]. It is logical to conclude that searching PubMed would lead many searchers to the publicly accessible PMC version of articles. Given that many researchers are also using Google Scholar as an access point to search for articles [12, 13], it is also worth considering that searching Google Scholar may lead searchers to the PMC version of an article. These easy and visible access points to the publicly accessible PMC version of articles may lead to greater readership and greater citation rates of these articles.

### Literature Review

PMC is a specialized open access repository. A number of studies have examined the impact of open online access on citation rates more generally. The findings of these studies, however, have been mixed. For example, a 2004 study examining the impact of open access in Philosophy, Political Science, Electrical and Electronic Engineering, and Mathematics found an increase in the mean citation rates of the open access articles compared to those that were not freely available online [14]. A 2006 research study of the Proceedings of the National Academy of Sciences journal articles found that the average number of citations, in the first 10–16 months of publication, to open access articles provided directly through the journal was higher than the number of citations to articles that were accessible only with a subscription to the journal [15]. A citation analysis comparing open access (OA) vs. non-open access articles in Nature Communications found that the OA content showed a small increase in citations but strikingly more usage in terms of views and downloads [16]. On the other hand, several studies have concluded that open access increases readership but not citation rates [17, 18].

Craig et al. conducted a comprehensive review of literature exploring the impact of open access on citation rates as of 2007 [19]. They noted that many of the studies exploring the impact of open access did not take confounding variables into account. One potential confounding variable is the timeframe in which the articles became openly available—it was possible that some versions of the articles were being made available prior to the release of the publisher version, and thus it may have been their early release, and not their open access format, that led to the appearance of increased citations. Craig et al. also highlight a potential problem with "selection bias" which is when an article is selected to be made openly accessible because the article itself is of high quality or potential impact. Thus, it is possible that the high quality of the article, and not the fact that the article was openly accessible, led to the higher citation rates.

### Methods

To determine the impact of the 2008 NIH Public Access Policy (PAP), NIH funded articles were examined for the publication years of 2006 and 2009, i.e., prior to vs. following the implementation of the 2008 policy. The year 2009 was the first full year where the 2008 NIH PAP was in place and, on the basis of the policy, any NIH funded articles from this year should have had a publicly accessible version available in PMC by sometime in 2010. Non-NIH funded articles published both before and after the policy was implemented were also examined, as an additional point of comparison.

A set of journal titles was identified from which to provide a collection of articles to examine. The list was developed from two sources:

1. the top 200 ranked journals based on impact factor in Journal Citation Reports (JCR) from the categories Behavioral Sciences, General and Internal Medicine, Research and Experimental Medicine, Nursing, and Obstetrics & Gynecology. Journals were obtained from a list where there was a high impact factor as this gave some indication of quality peer review and thus suggested there was a certain level of quality expected in the articles.
2. Journals on the Abridged Index Medicus (AIM) list. AIM is a list of approximately 120 core clinical English language journals developed and maintained by the National Library of Medicine [20].

There was some overlap between journals on the two lists. Using a collection of articles from a specified set of journals allows for uniformity between the articles examined within a journal. Articles should have a similar scope of peer review, quality and topical areas, and a relatively similar impact factor for a journal across the timespan studied. Journals were excluded from the list if:

1. They were not indexed in Scopus. Scopus, an abstract and citation database produced by Elsevier, was the database selected to provide the number of cited references for each article included in the study. Scopus has been reported to be the most comprehensive article-level index of scholarly articles [21].
2. They were an open access journal or became freely accessible after an embargo period (e.g. freemedicaljournals.com, http://www.ncbi.nlm.nih.gov/pmc/).
3. None of the 2009 articles in a journal were archived in PubMedCentral. The inclusion requirement for a journal to have at least one article archived in PMC in 2009 ensured there were articles for comparison to other non-PMC archived articles in the journal.
4. Less than 2% of the articles in a journal were funded by the NIH. Requiring a certain percentage of articles to be funded by the NIH ensured there were at least some articles in the NIH funded group for each journal included in the study.

The citation information and cited reference counts of all the articles published in 2006 and 2009 for the journals included in the study were obtained from Scopus. Scopus searches were limited to article or review articles to eliminate items such as letters and editorials. Further searches were performed in PubMed using “publication type” information to identify and remove non-research items such as editorials, letters, bibliographies, and retraction notices from the data.

To determine which of these articles were included as full-text in PMC, each journal was searched in PubMed. The “free full-text” filter in PubMed was used to indicate freely accessible articles, which includes articles in PMC. Each 2006 and 2009 article archived in PMC was examined in PMC to determine the number of months after the official date of publication the article became available in PMC. Articles published in 2006 but not available in PMC by January 2010 or later were treated as non-PMC articles; similarly, articles published in 2009 but not available in PMC by January 2013 were also treated as non-PMC articles. It is unlikely these articles would be cited in another 2013 publication as a result of their 2013 availability in PMC. One study found that on average, only 2% of citations an article receives occurs within the first year of the article being published [22].

The “Research Support, N.I.H. Extramural” and “Research Support, N.I.H. Intramural” filters in PubMed were used to identify articles funded by the NIH. If an article was available full-text in PMC but did not appear with NIH funding when the filters were used, the PubMed abstract view was selected to view the "Grant Support" provided for the article. Articles with NIH funding listed in the grant support field were also considered to have NIH funding. Since the focus of the study was on the impact of the NIH PAP, articles in PMC that had no indication of NIH funding in PubMed were dropped from the study to avoid confounding the results. Such articles were likely funded by a non-U.S. source that also required deposit in PMC (PMC Canada, Europe PMC). Citing data was captured yearly and cumulatively for a five-year period. For articles published in 2006, citing data from 2006–2010 were recorded. For articles published in 2009, citing data from 2009–2013 were recorded. Citations were then categorized into independent groups of journal articles based on year published, NIH funding, and PMC availability.

The majority of the journals that were excluded from the initial list of journals were dropped because they met exclusion criteria 2 or 3 as described above. An additional 13 journals were dropped once the data was collected and it was determined they met exclusion criterion 4. A total of 45,716 articles published in 2006 and 2009 from 122 journals were included in the study.

## Results

### Analysis of 2006 articles

**Fig 1:**
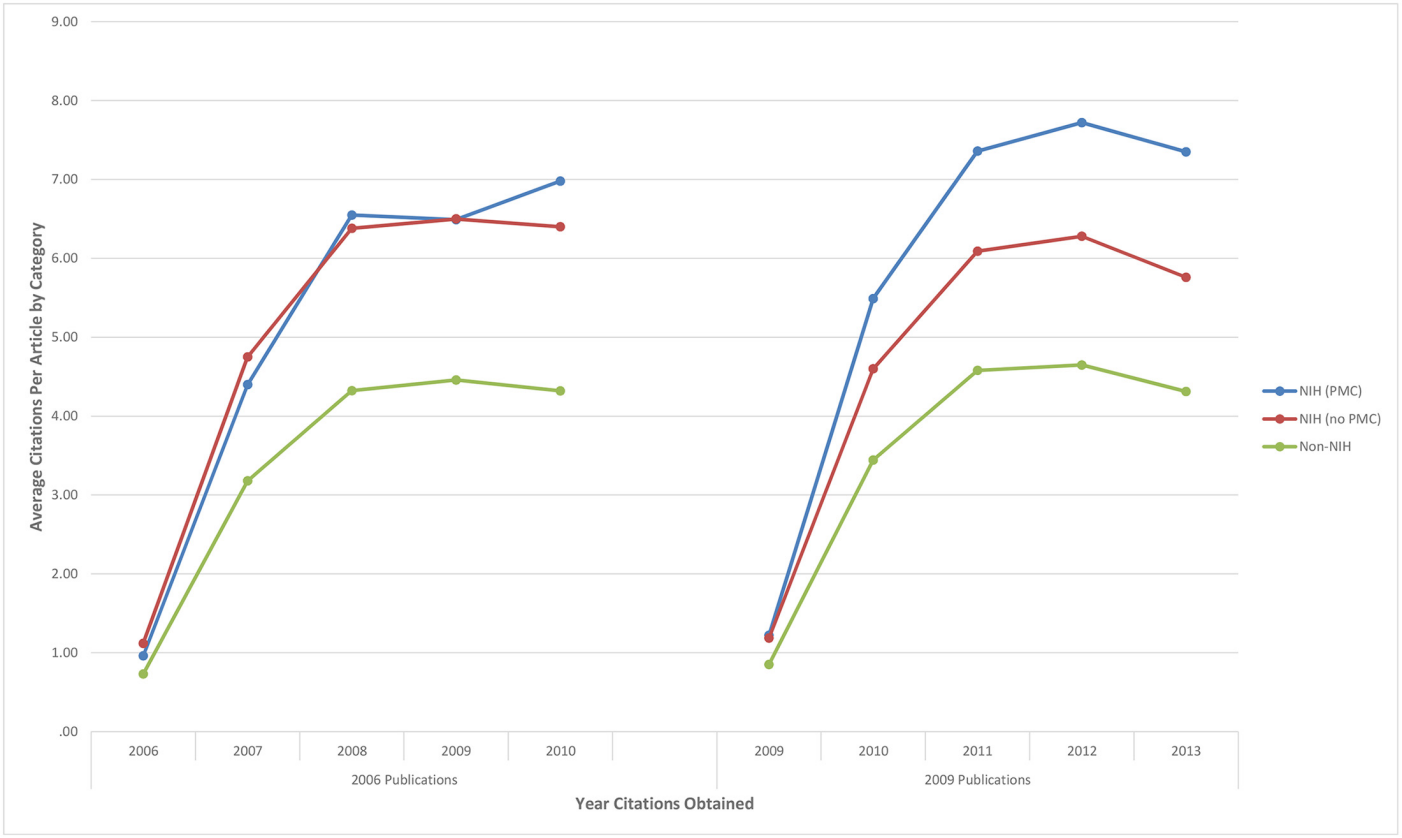
Average cites per article over a 5 year period by NIH funding status and PMC status for 2006 & 2009 publications. Citation data were collected in March 2014, when most (but not all) 2013 citation data were available in Scopus. The 2013 citation data are thus a slight underestimate of the true value, but this does not appear to have biased our PMC vs. non-PMC comparison.

About 15.8% of the 2006 articles included in the study were funded by the NIH, of which only 9.4% of the NIH-funded articles were deposited in PMC by January 2010. Mean cumulative citation rates and the number of articles appearing in each category for 2006 are shown in Table 1. Fig 1 shows the mean citation rates of 2006 NIH funded articles in PMC, compared to the NIH funded articles not in PMC, over the 5 year period examined. Over a 5 year period (within factor), the cumulative cited references for the 2006 NIH funded articles archived in PMC were compared to the cumulative cited references for the 2006 NIH funded articles that were not archived in PMC (between factor). There was no statistically significant difference between the mean citation rates of NIH funded articles in PMC compared to those NIH funded articles not in PMC, neither yearly nor cumulatively over the 5 year period examined (repeated measures ANOVA *F(1*, *3576)* = .040, *p* = .842, η_p_^2^ = .0001). An ANOVA blocked for journal was also conducted to directly compare PMC vs. non-PMC articles published in the same journal to control for possible citation, quality, and discipline differences. Differences in the mean cumulative citation rates between the two groups remained non-significant (*F*(1,3142) = .655, *p* = .418).

**Table 1:** Summary of Article Categories and Mean Citation Rates after a 5 Year Period by NIH Funding, PMC Status and Year.

	2006					2009				
	No NIH funding	NIH Funded			2006 Total	No NIH funding	NIH Funded			2009 Total
		NIH/ No PMC	NIH/ PMC	NIH Total			NIH/ No PMC	NIH/ PMC	NIH Total	
N	19,142	3241	337	3578	22,720	18,614	1263	3119	4382	22,996
Mean Cumulative Citation Rates (after 5 Years)	**17.01**	**25.14**	**25.37**	**25.17**	**18.30**	**17.84**	**23.91**	**29.14**	**27.63**	**19.70**
±SD	±31.2	±41.3	±28.7	±40.3	±32.9	±33.4	±41.3	±54.3	±50.9	±37.6

Of the 2006 NIH funded articles deposited in PMC, fewer than half were deposited within the first 12 months after publication (3.3% within 1 month, 12.8% within 6 months, and 28% in 7–12 months). An additional 8% were available between 12–24 months and 25% within 37–48 months. As deposit in PMC was voluntary at this time, 90.6% of 2006 NIH funded articles were not archived in PMC by January 2010.

### Analysis of 2009 articles

About 19.1% of the 2009 articles included in the study were funded by the NIH, of which 72.4% of NIH-funded articles were deposited in PMC. Mean cumulative citation rates and the number of articles appearing in each category for 2009 are shown in Table 1. Fig 1 shows the mean citation rates of 2009 NIH funded articles in PMC, compared to those not in PMC, over the 5 year period examined. The 2009 NIH funded articles in PMC are cited significantly more than those NIH funded articles not in PMC, in years 2–5. Over a 5 year period, the cumulative cited references for the 2009 NIH funded articles archived in PMC were compared to the cumulative cited references for the 2009 NIH funded articles that were not archived in PMC. There was a statistically significant difference between the mean citation rates of NIH funded articles in PMC compared to those not in PMC (repeated measures ANOVA *F(1*,*4380)* = 8.924, *p*< 0.003, η_p_^2^ = .002.). Five years after publication, the PMC articles were cumulatively cited 26% more on average than non-PMC articles (29.14 times vs. 23.91 times). When an ANOVA analysis blocked for journal was conducted, the difference between the PMC vs. non-PMC articles in the same journal was still statistically significant (*F*(1,4322) = 6.406, *p* = .011, η_p_^2^ = .002.).

Of the 2009 NIH funded articles that became available in PMC by January 2013, 83% were available in PMC within the first 12 months (1.5% within 1 month, 8.2% within 6 months, and 73.3% within 7–12 months). An additional 9.5% were available between 12 and 24 months. However, 27.6% of 2009 NIH funded articles remained non-compliant by January 2013.

## Discussion

We found that NIH funded articles published in 2009, and made publicly accessible in PubMed Central (PMC), are cited significantly (26%) more, on average, than comparable articles published in the same journal but not deposited in PMC. In contrast, no such difference was observed for NIH funded articles published in 2006. What changed between 2006 and 2009 that might explain why PMC provided a significant boost to scholarly impact as of 2009 but not earlier?

Perhaps the most plausible explanation is that the NIH Public Access Policy (PAP) was implemented in 2008, leading to rapid acceptance of PMC as a mandated repository for NIH funded articles, with the high visibility that this entails. An additional factor may be the fact that 2006 articles were deposited into PMC after relatively long delays compared to 2009 articles. This is likely to have impacted their subsequent citations, as shown by the observed correlation between PMC archiving dates and cumulative cites at 5 years. This correlation was significant for 2006 PMC articles (Pearson *r* = -0.135, *n* = 337, *p* = 0.013) but not for 2009 PMC articles (Pearson *r* = -.001, *n* = 2740, *p* = .963).

Yet another factor may be the increase in access to PMC articles that is provided through Google Scholar. A 2008 article reported that "only about a quarter of the open access PubMed Central (PMC) items are directly available in Google Scholar" [23]; in contrast, PMC is fully accessible through Google Scholar today. Further, researchers increasingly report using Google Scholar as one of their primary search tools [12, 13]. In fact, PMC currently gets more referrals from Google (not specifically Google Scholar) than from PubMed [24].

It is clear that the visibility of PMC (across all search engines) continues to increase significantly from year to year, in terms of number of online queries per weekday (>2 million articles), which is growing at a higher rate than the growth of articles in PMC itself [24]. Increased user views may not necessarily translate into increased citations, but this provides a plausible mechanism for the citation effects that we observed.

We suggest that analyses of the scholarly impact of open access should take into account the rapidly evolving landscape of scientific publishing as well as the changing tastes and behaviors of researchers. For example, after the introduction of online journals, use of the print collections decreased, even for journals available only in print format to the researchers [25, 26]. Other studies found that researchers cited more from online journals and less from the journals available only in print, once online journals were introduced [27, 28].

This study also found that NIH funded articles are cited more, on average, than non-NIH funded articles. It is possible the non-NIH funded articles included in this study represented a broader spectrum of publication types than may have been represented with the NIH funded articles. It is also possible the quality of the projects selected by the NIH for funding lead to quality research and publications, which in turn accounts for the higher citation rates of these publications over the non-NIH funded articles.

### Limitations

Our 2009 data are unlikely to have been confounded by factors discussed by Craig et al [19] such as different publication release dates (since the vast majority of articles were available in PMC within the first year of publication) or intrinsic article quality (since deposition into PMC was legally mandated and broad-based, and since we compared articles that were published in the same journals). Nevertheless, there is likely to be some intrinsic difference(s) between 2009 NIH funded articles deposited vs. not deposited in PMC, that we have not defined precisely, and which deserve further analysis. Because there were many articles that PubMed identified only as “Journal Article” for publication type, it was unknown what study design these articles would have employed without examining each article. The quality of an article undoubtedly plays a role in the number of times an article is cited. We compared NIH funded articles published in the same journal which is the best assurance that they met similar quality standards and had similar visibility to readers.

We did note that 2009 NIH funded articles deposited in PMC had, on average, a higher number of authors per article ($\bar{x}=6.14$) compared to non-PMC articles ($\bar{x}=5.68$) (*t* = 3.22, *p* = .001). This suggests that the PMC set of articles might include a higher proportion of collaborative studies, or may represent a higher proportion of publication types that tend to have many authors (e.g., clinical trials). There was a small but significant correlation between the number of authors on a paper and its cumulative cites at 5 years, both for all 2006 NIH funded papers (*r* = 0.17, *n* = 3578, *p*< 0.0001) and for all 2009 NIH funded papers (*r* = 0.16, *n* = 4382, *p*< 0.0001). However, a multiple regression of the 2009 NIH funded publications (holding the number of authors constant) showed that there was still a significant independent effect of PMC archiving on the number of citations an article received (*t* = 2.615, *p* = .009). Thus, the apparent effect of PMC on citations cannot be entirely explained by intrinsic differences between PMC and non-PMC articles that are associated with numbers of authors per article.

This study examined a set of articles from a small (but high-quality) subset of journals from a vast set of potential journals. Full sets of journals from specific disciplines were not examined, so it was not possible to assess discipline specific differences. A follow-up study examining larger sets of journals from specific disciplines would be valuable to determine if there are field specific differences related to the citation impact of the NIH Public Access Policy.

## Conclusion

This study illustrates a positive impact of the 2008 NIH Public Access Policy. Articles that are openly accessible in PubMed Central are often cited more than articles published in the same journals, but not openly accessible. This increase in scholarly impact is important to researchers and their institutions, to NIH, and to the public. This is a strong argument for expanding legislation to other federal agencies making information more accessible.

## References

[1] National Institutes of Health, Department of Health & Human Services. (2005) Policy on enhancing public access to archived publications resulting from NIH-funded research. Notice Number: NOT-OD-05-022. Available: http://grants.nih.gov/grants/guide/notice-files/NOT-OD-05-022.html. Accessed 9 September 2014.1.

[2] National Institutes of Health, Department of Health & Human Services (2014). Frequently asked questions about the NIH Public Access Policy: what is the NIH Public Access Policy? Available: http://publicaccess.nih.gov/faq.htm#753. Accessed 9 September 2014.

[3] National Institutes of Health, Department of Health & Human Services (2014). National Institutes of Health public access: Overview. Available: http://publicaccess.nih.gov/. Accessed 7 May 2014.

[4] National Institutes of Health, Department of Health & Human Services. (2013) NIH public access policy details. Available: http://publicaccess.nih.gov/policy.htm. Accessed 7 May 2014.

[5] National Library of Medicine, National Institutes of Health. PMC overview. Available http://www.ncbi.nlm.nih.gov/pmc/about/intro. Accessed 11 May 2014.

[6] U.S. National Library of Medicine, National Institutes of Health. (2014) PMC. Available http://www.ncbi.nlm.nih.gov/pmc/. Accessed 27 April 2015.

[7] National Institutes of Health, Department of Health & Human Services (2014). Submission methods. Available: http://publicaccess.nih.gov/submit_process.htm. Accessed 9 September 2014.

[8] National Center for Biotechnology Information, National Institutes of Health. (2014) NIHMS statistics: monthly aggregate submission statistics. Available: http://www.nihms.nih.gov/stats/. Accessed 11 May 2014.

[9] Cooper P., Gibb S, Thakur N., Trawick B. (2013) Changes to the NIH Public Access Policy and the implications. Available: http://grants.nih.gov/grants/webinar_docs/webinar_20130115.htm. Accessed 7 May 2014.

[10] National Center for Biotechnology Information, National Library of Medicine. PMC FAQs. Available: http://www.ncbi.nlm.nih.gov/pmc/about/faq/. Accessed 9 September 2014.

[11] National Library of Medicine, National Institutes of Health. (2013) FAQ: PubMed. Available: http://www.nlm.nih.gov/services/pubmed_searches.html. Accessed 12 May 2014.

[12] De Groote SL, Shultz M, Blecic DB. (2014) Information seeking behavior and the use of online resources: a snapshot of current health sciences faculty. Journal of the Medical Library Association 102: 169–176 10.3163/1536-5050.102.3.006 25031557 PMC4076125

[13] Ollé C, Borrego Á. (2010) A qualitative study of the impact of electronic journals on scholarly information behavior. Library & Information Science Research 32: 221–228. 10.1016/j.lisr.2010.02.002

[14] Antelman K. (2004) Do open-access articles have a greater research impact? College & Research Libraries 65: 372–382.

[15] Eysenbach G. (2006) Citation advantage of open access articles. PLoS Biology 4: e157 06-PLBI-RA-0134R2 [pii]. 16683865 10.1371/journal.pbio.0040157 PMC1459247

[16] Research Information Network. Nature Communications: Citation Analysis. 2014. Available: http://www.nature.com/press_releases/ncomms-report2014.pdf. Accessed 1 September 2014.

[17] Davis PM, Lewenstein BV, Simon DH, Booth JG, Connolly MJ. (2008) Open access publishing, article downloads, and citations: randomised controlled trial. BMJ 337: a568 10.1136/bmj.a568 18669565 PMC2492576

[18] Davis PM. (2011) Open access, readership, citations: a randomized controlled trial of scientific journal publishing. FASEB Journal 25: 2129–2134. 10.1096/fj.11-183988 21450907

[19] Craig ID, Plume AM, McVeigh ME, Pringle J, Amin M. (2007) Do open access articles have greater citation impact?: A critical review of the literature. Journal of Informetrics 1: 239–248.

[20] U.S. National Library of Medicine, National Institutes of Health. (2013) Abridged Index Medicus (AIM or "core clinical") journal titles. Available: https://www.nlm.nih.gov/bsd/aim.html. Accessed 7 May 2014.

[21] Laakso M, Bjork BC. (2012) Anatomy of open access publishing: A study of longitudinal development and internal structure. BMC Med 10: 124-7015-10-124. 10.1186/1741-7015-10-124 23088823 PMC3478161

[22] De Groote SL. (2008) Citation patterns of online and print journals in the digital age. Journal of the Medical Library Association 96: 362–369. 10.3163/1536-5050.96.4.012 18974814 PMC2568853

[23] Jacsó P. (2008) "Google Scholar revisited", Online Information Review, 32: 102–114

[24] NLM/NCBI List—PubmedCentral. Correspondence with PubMedCentral Help via Monica Romiti, NCBI User Services. Received 13 Nov 2014.

[25] De Groote SL, Dorsch JL. (2001) Online journals: impact on print journal usage. Bulletin of the Medical Library Association 89: 372–378. 11837259 PMC57966

[26] Vaughan KTL. (2003) Changing use patterns of print journals in the digital age: Impacts of electronic equivalents on print chemistry journal use. Journal of the American Society for Information Science & Technology 54: 1149–1152. 10.1002/asi.10319

[27] De Groote SL. (2008) De Groote, S. L. (2008). Citation patterns of online and print journals in the digital age. Journal of the Medical Library Association 96: 362–369. 10.3163/1536-5050.96.4.012 18974814 PMC2568853

[28] De Groote SL, Barrett FA. (2010) Impact of online journals on citation patterns of dentistry, nursing, and pharmacy faculty. Journal of the Medical Library Association 98: 305–308. 10.3163/1536-5050.98.4.008 20936070 PMC2947137

